# Next-Generation Sequencing Identifies Extended HLA Class I and II Haplotypes Associated With Early-Onset and Late-Onset Myasthenia Gravis in Italian, Norwegian, and Swedish Populations

**DOI:** 10.3389/fimmu.2021.667336

**Published:** 2021-06-07

**Authors:** Lisa E. Creary, Sridevi Gangavarapu, Stacy J. Caillier, Paola Cavalcante, Rita Frangiamore, Benedicte A. Lie, Mats Bengtsson, Hanne Flinstad Harbo, Susanna Brauner, Jill A. Hollenbach, Jorge R. Oksenberg, Pia Bernasconi, Angelina Hatlø Maniaol, Lennart Hammarström, Renato Mantegazza, Marcelo A. Fernández-Viña

**Affiliations:** ^1^ Department of Pathology, Stanford University School of Medicine, Palo Alto, CA, United States; ^2^ Histocompatibility, Immunogenetics and Disease Profiling Laboratory, Stanford Blood Center, Palo Alto, CA, United States; ^3^ Department of Neurology, School of Medicine, University of California San Francisco, San Francisco, CA, United States; ^4^ Neurology IV Unit Neuroimmunology and Neuromuscular Diseases, Fondazione I.R.C.C.S Istituto Neurologico Carlo Besta (INCB), Milan, Italy; ^5^ Department of Immunology and Transfusion Medicine, Oslo University Hospital, Oslo, Norway; ^6^ Institute of Clinical Medicine, University of Oslo, Oslo, Norway; ^7^ Department of Medical Genetics, University of Oslo and Oslo University Hospital, Oslo, Norway; ^8^ Department of Immunology, Genetics and Pathology (IGP), Rudbeck Laboratory, Uppsala University and University Hospital, Uppsala, Sweden; ^9^ Department of Neurology, Oslo University Hospital and University of Oslo, Oslo, Norway; ^10^ Department of Clinical Neuroscience, Karolinska Institutet, Stockholm, Sweden; ^11^ Department of Neurology, Oslo University Hospital, Oslo, Norway; ^12^ The Department of Laboratory Medicine, Karolinska Institutet, Stockholm, Sweden; ^13^ Department of Clinical Research and Innovation, Fondazione I.R.C.C.S Istituto Neurologico Carlo Besta (INCB), Milan, Italy

**Keywords:** myasthenia gravis, human leukocyte antigen, next-generation sequencing, European, susceptibility, protection

## Abstract

Genetic susceptibility to myasthenia gravis (MG) associates with specific HLA alleles and haplotypes at the class I and II regions in various populations. Previous studies have only examined alleles at a limited number of HLA loci that defined only broad serotypes or alleles defined at the protein sequence level. Consequently, genetic variants in noncoding and untranslated HLA gene segments have not been fully explored but could also be important determinants for MG. To gain further insight into the role of HLA in MG, we applied next-generation sequencing to analyze sequence variation at eleven HLA genes in early-onset (EO) and late-onset (LO) non-thymomatous MG patients positive for the acetylcholine receptor (AChR) antibodies and ethnically matched controls from Italy, Norway, and Sweden. For all three populations, alleles and haplotype blocks present on the ancestral haplotype AH8.1 were associated with risk in AChR-EOMG patients. HLA-B*08:01:01:01 was the dominant risk allele in Italians (OR = 3.28, *P* = 1.83E−05), Norwegians (OR = 3.52, *P* = 4.41E−16), and in Swedes HLA-B*08:01 was the primary risk allele (OR = 4.24, P <2.2E-16). Protective alleles and haplotype blocks were identified on the *HLA-DRB7*, and *HLA-DRB13.1* class II haplotypes in Italians and Norwegians, whereas in Swedes *HLA-DRB7* exhibited the main protective effect. For AChR-LOMG patients, the *HLA-DRB15.1* haplotype and associated alleles were significantly associated with susceptibility in all groups. The *HLA-DR13–HLA-DR–HLA-DQ* haplotype was associated with protection in all AChR-LOMG groups. This study has confirmed and extended previous findings that the immunogenetic predisposition profiles for EOMG and LOMG are distinct. In addition, the results are consistent with a role for non-coding HLA genetic variants in the pathogenesis of MG.

## Introduction

Myasthenia gravis (MG) is a rare antibody-mediated, T-cell dependent autoimmune disorder of the neuromuscular junction (NMJ) characterized by fluctuating muscle weakness and abnormal fatigability (1, 2). The global prevalence of MG ranges from 40 to 180 per million with an estimated annual incidence of 1.74 to 12 cases per million persons (3, 4). MG occurs across all ancestral groups and affects individuals of all ages and both sexes, although several studies have shown that MG exhibits a binomial distribution based on age and sex; MG is frequently observed in younger women aged less than 40 years old and older men over 60 years old (4).

In the majority of patients, ~85–90%, MG is a result of pathogenic autoantibodies targeting the nicotinic acetylcholine receptors (AChRs) located at the postsynaptic membrane of the NMJ (5–7). Anti-AChR autoantibodies predominantly consist of immunoglobulin subclasses IgG1 and IgG3, which are potent activators of complement resulting in membrane attack, impaired synaptic signal transmission, and muscle damage that manifests as skeletal muscle weakness (8). In addition, CD4+ T helper cells and the cytokines they secrete play important roles in the pathogenesis of MG. Rare forms of MG are due to autoantibodies targeting other postsynaptic membrane components such as the muscle-specific kinase (MuSK) protein, which is essential for NMJ formation and clustering of AChRs, and the lipoprotein receptor-related protein 4 (LRP4) a receptor that binds to neuronal agrin to activate MuSK (5, 9). Double-seronegative MG cases (meaning negative for AChR and MuSK antibodies) may have other antibodies targeting NMJ proteins (such as LRP4, Agrin, and Cortactin), but further studies are needed to determine their relevance for disease mechanism (5, 9, 10). The MG autoantibodies are important diagnostic factors and are used along with the clinical presentation (ocular *vs*. generalized), thymus pathology, and age of onset for classifying MG patients and to help guide treatment (3). The demarcation between early-onset MG (EOMG) and late-onset MG (LOMG) is not well-defined, but most studies agree than EOMG occurs at <50 years, and LOMG is >50 years old (3, 11). These two subtypes of MG differ in terms of sex affected and the associated susceptibility genes; EOMG is common in women with a hyperplastic thymus, whereas LOMG mostly afflicts men who have normal or atrophic thymus. The different MG subgroups clearly indicate that MG is a heterogeneous disease, suggesting that many predisposing factors are involved in determining disease pathogenesis. The disease etiology in MG is multifactorial involving a complex interplay of environmental and genetics factors. Familial cases of MG have been reported over the years, implicating the role of genetic factors in MG risk (12–16). Furthermore, a twin study examining MG heritability showed that the concordance rate between monozygotic twins (35%) was greater than dizygotic twins (3–4%), further underscoring the notion of a genetic predisposition (17). However, the incomplete concordance in monozygotic twins also suggests the contribution of a large environmental component to MG risk. The strongest genetic predictors of MG risk are the human leukocyte antigen (HLA) genes (18) located on chromosome 6p21.31. The associated HLA alleles vary according to the different subgroups of MG. Specifically, the *HLA-A1-B8-DR3-DQ2* ancestral haplotype, also referred to as AH8.1 (19), or specific alleles which lie on this haplotype have been found to be strongly associated with AChR-MG and EOMG in several European populations (20–24). The *HLA-B7-DR2* haplotype and the HLA-DRB1*15:01 allele were found to be associated with LOMG in two cohorts (25, 26). In a recent retrospective study performed in Italian MG patients HLA-DQB1*05:01 was associated with thymoma, and the HLA-DRB1*16~HLA-DQB1*05:02 haplotype with non-thymomatous AChR-MG with >60 years onset (27). These studies highlight the genetic heterogeneity in HLA alleles predisposing to various groups of MG among different populations. However, previous studies were limited by the small number of HLA genes interrogated in a single study, and restricted coverage of genes. Limited sequence coverage of HLA genes means that allele and phase ambiguities are likely to be present, thereby compromising the accuracy of the alleles and haplotypes defined. Therefore, re-examination of HLA associations with MG using extended coverage and advanced molecular typing methods is warranted. Next-generation sequencing (NGS) is an attractive option for variant characterization of HLA genes due to its comparatively low cost, high-accuracy and unbiased variant discovery. NGS of extended HLA gene segments has been applied successfully in population-based and trait-association studies to identify allelic variants in non-coding regions, and to achieve mostly unambiguous HLA genotyping (28–31). In this study, we report the analyses of HLA alleles and haplotypes, defined using high-resolution NGS, associated with susceptibility and protection to EOMG and LOMG in Italian, Norwegian, and Swedish cohorts.

## Subjects and Methods

### Study Populations

The study populations consisted of MG patients and healthy controls (HC) recruited from Italy (MG = 354, 219 females; HC = 250, 90 females), Norway (MG = 412, 252 females; HC = 500, 319 females), and Sweden (MG = 339, 202 females; HC = 2,120, sex unknown). All participants provided written informed consent at the site of study recruitment and arrived de-identified to the genotyping and analysis laboratories (Stanford University and the University of California San Francisco). All subjects included in the analyses were self-described white European. There was not a requirement for ancestors living in Italy for a certain number of generations, however, patients and controls were reported to be of Italian descent. White Norwegians was classified according to the birth country and ethnicity of the parent. The Swedish cohort is a single-center consecutively collected cohort of MG patients either treated or reviewed for second opinion at the Karolinska University Hospital in Stockholm, Sweden. Self-reported information on ancestry was collected at inclusion. There was no requirement for ancestors having lived in Sweden for a certain number of generations.

### HLA Genotyping

HLA typing was performed on genomic DNA extracted from blood samples using local protocols such as the salting-out method or the standard phenol/chloroform method. For all DNA samples with the exception of Swedish HC samples which were typed previously by mid-resolution Sanger sequencing, were retrospectively typed at high-resolution for class I (*HLA-A*, *HLA-C*, *HLA-B*) and class II (*HLA-DRB1*, *HLA-DRB3*, *HLA-DRB4*, *HLA-DRB5*, *HLA-DQA1*, *HLA-DQB1, HLA-DPA1, HLA-DPB1*) HLA loci using the MIA FORA NGS high-throughput (HT) semi-automated typing protocol (Immucor, Inc., Norcross, GA, USA) and Illumina MiniSeq instruments (Illumina, Inc., San Diego, CA, USA). All sample testing including sequence data analysis and HLA genotype assignment using the MIA FORA FLEX v3.0 alignment software (Immucor, Norcross, GA, USA), were performed according to previously published methods (28).

### Standardization of STR Ambiguities

The MIA FORA software permits detailed examination of all sequence segments, as well as unambiguous allele assignment, with the exception of short tandem repeat (STR) enriched regions located within introns of some class II genes. These low complexity regions consist of ~1–6 bp nucleotide units that are repeated numerous times and cannot be enumerated accurately by the sequencing methodology. In order to standardize alleles that are indistinguishable due to STRs, alleles were assigned to groups and were given the suffix SG (denoting STR Group) to the lowest numbered allele in that group (28, 30). For example, allele HLA-DRB1*03:01:01:01SG denotes the HLA-DRB1*03:01:01:01 and HLA-DRB1*03:01:01:02 STR ambiguous group. Details of HLA ambiguities are shown in **Supplementary Table 1**.

### Statistical Analyses

Allele carrier frequencies were determined by direct counting from the sequencing results and were calculated by dividing the number of times a specific allele (either at the homozygous or heterozygous state) is observed in a population by the total number of copies of all alleles at that particular genetic locus in the population. The Bridging Immuno Genomic Data Analysis Workflow Gaps (BIGDAWG) software package v1.8 (32) was used to perform case-control phenotype association analyses. Association analyses were performed using chi-square (χ2) testing. Haplotypes frequencies were estimated using the R ‘haplo.stats’ package to run the expectation maximization (EM) algorithm in the BIGDAWG package. A tilde symbol “~” is used to show alleles located or shared on a specific haplotype block. Calculation of haplotype/allele confidence interval, odds ratio, and *P*-values were performed using the R epicalc package run in BIGDAWG. The effect sizes of HLA alleles and haplotypes on MG risk were measured by odds ratio (OR) with 95% confidence intervals (CI), and associated probability (*P)* values were derived from a two-tailed Fischer’s exact test or a Pearson’s chi-squared test when appropriate. Correction of nominal *P*-values for multiple testing was applied as follows. First the Bonferroni correction method was used to correct *P*-values generated from tests of overall (locus-level) heterogeneity at the locus. *P*-values less than 0.05 (α) were considered statistically significant. If the adjusted locus-level heterogeneity *P*-value at a particular locus was statistically significant allele-level association *P*-values were not corrected for multiple comparison, However, if the adjusted locus-level heterogeneity *P*-value at a locus was not statistically significant allele-level association *P*-values were corrected for multiple testing (i.e. multiplying the *P*-value by the number of identified alleles at each locus) using the Bonferroni method. Stratification analysis was applied to the primary risk alleles (i.e. the allele(s) with the strongest effect) to detect secondary alleles and haplotypes associated with the disease. Due to the small number of ocular patients from each population (ranging from 13 to 19 in the EOMG group, and three to 30 in the LOMG group), ocular data from all three groups were pooled together then analyzed. All calculated *P*-values were two-sided and *P*-values less than 0.05 (α) were considered statistically significant. Standardized linkage disequilibrium (LD) measures were computed using *D*’ (33) and the W_n_ statistic (34) at the locus-level and *D*’_ij_ for alleles at two loci implemented in the PyPop software (35). In control subjects allele frequencies at all loci were tested for deviations from Hardy–Weinberg equilibrium (HWE) proportions using the exact method of Guo and Thompson (36). Analyses were performed using the *R* statistical program version 3.5.0 and SPSS version 21 software package (IBM SPSS Inc., Chicago, IL, USA).

## Results

### Demographics of Cohorts

MG datasets were categorized according to age of onset; early-onset MG (EOMG) age of <50 years, and late-onset MG (LOMG) age of >50. In addition, the cohorts were summarized according to ocular and generalized MG subtypes, autoantibody profile, and thymoma cases (**Table 1**). We first sought to determine whether gender influenced any of the clinical features described. We found that the distribution of male and females were significantly different among EOMG and LOMG patients in all three groups (*P <*0.001). There was a predominance of female cases in the EOMG group, ranging from 77.6% in the Italian cohort to 79.0% in Norwegians and 80.4% in Swedish patients. In contrast, LOMG was more common in men, occurring at 66.7% in Italians, 62.7% in Norwegians, and 61.1% in Swedes. These finding are in keeping with previous reports of sex differences in MG by age of onset. Similarly, females were prominent in the anti-AChR-Ab seropositive group in all three cohorts, ranging from 57 to 60%. Thymoma was slightly more common in women although the numbers are small. We restricted the analyses to patients that were non-thymomatous and antibody positive for the AChR since the dataset of the rarer antibody positive subsets were too small to analyze and make any inferences.

**Table 1:** Characteristics of myasthenia gravis patient cohorts.

Characteristic	Italian		Norwegian		Swedish	
	No. patients (women/men)	*P*	No. patients (women/men)	*P*	No. patients (women/men)	*P*
Total MG patients, n	354 (219/135)	<0.001^c^	412 (252/160)	0.413^c^	339 (202/137)	n/a^c^
Total HC, n	250 (90/160)		500 (319/181)		2120 (uk/uk)	
Age at disease onset						
<50 years (EOMG)^a^	165 (128/37)	<0.001^d^	214 (169/45)	<0.001^d^	143 (115/28)	<0.001^d^
Ocular MG type	25 (12/13)		28 (20/8)		29 (18/11)	
Generalized MG type	124 (105/19)		184 (147/37)		114 (97/17)	
>50 years (LOMG)^b^	93 (31/62)		150 (56/94)		149 (58/91)	
Ocular MG type	7 (2/5)		40 (10/30)		40 (11/29)	
Generalized MG type	78 (25/53)		109 (46/63)		109 (47/62)	
Autoantibody profile						
Anti-AChR positive	241 (137/104)		327 (194/133)		256 (153/103)	
Anti-MuSK positive	27 (23/4)		7 (6/1)		2 (1/1)	

MG, myasthenia gravis; HC, healthy controls; ^a^EOMG, early-onset myasthenia gravis and non-thymomatous; ^b^LOMG, late-onset myasthenia gravis and non-thymomatous; Anti-AChR, acetylcholine receptor autoantibodies; Anti-MuSK, muscle specific tyrosine kinase autoantibodies; P, probability values for the chi-square statistic (significant at P < 0.05) comparing the gender distribution ^c^between patients and controls, and ^d^EOMG and LOMG patients; u/k, unknown.

### Locus-Level Heterogeneity in AChR-EOMG Patients

We tested eleven HLA loci characterized at maximum resolution in the Italian and Norwegian case-control cohorts and six loci in the Swedish group for allelic and haplotype heterogeneity and association with MG risk and protection. The Swedish data was examined at 2-field allelic resolution for six HLA genes (*HLA-A*, *HLA-C*, *HLA-B*, *HLA-DRB1, HLA-DQB1*, and *HLA-DPB1*) because the control sample used was previously genotyped for only these loci using mid-resolution molecular methods. For all three populations, HLA heterogeneity between AChR-EOMG non-thymomatous cases and controls were analyzed at the overall locus-level, the results of which are shown in **Supplementary Table 2**. In the Italian cohort following adjustment for multiple testing, the majority of loci and haplotypes were significant with the exception of *HLA-A*, *HLA-C*, *HLA-DPA1*, *HLA-DPB1*, and *HLA-DPA1~HLA-DPB1*. Similarly, in the Norwegian group most of the loci survived multiple testing and only the *HLA-DP* loci and haplotype results were not significant. In the Swedish cohort all loci with the exception of *HLA-DPB1* were statistically significant. The significant results indicate differences in the distribution of HLA alleles between cases and controls.

### The HLA-B*08~HLA-DR3~HLA-DQA1*05~HLA-DQB1*02 Haplotype Is Associated With AChR-EOMG Risk

The distribution of haplotype blocks encompassing two to eleven loci in cases and controls were compared. For all three case-control populations the strongest associations were identified between AChR-EOMG susceptibility and haplotype blocks present on the conserved ancestral haplotype (AH) 8.1 found in populations originating from Northern and Western Europe (**Tables 2**–**4**). Specifically, the risk haplotype blocks included various combinations of class I blocks (e.g. HLA-C*07:01:01:01~HLA-B*08:01:01:01), class II blocks (HLA-DRB1*03:01:01:01SG~HLA-DQA1*05:01:01:02), and class I and II blocks (HLA-B*08:01:01:01~HLA-DRB1*03:01:01:01SG). A similar pattern was observed in the Swedish population that was analyzed at 2-field allelic resolution. These results indicate that both class I and II regions of AH8.1 influence AChR-EOMG risk. It is noteworthy that the shorter haplotype blocks bearing HLA-B*08:01:01:01 have a greater effect on disease risk, compared to haplotype blocks without HLA- B*08:01:01:01, suggesting that HLA-B*08:01:01:01 is the dominant risk allele.

**Table 2:** Significant associations of HLA alleles and haplotypes in Italian EOMG AChR-antibody positive cases and controls ranked by odds ratios.

HLA Locus	HLA alleles/haplotypes	EOMG Cases		Controls		95% CIOR	95% CI	*P*-value	*P*-adj	Effect
		Count	Fq	Count	Fq					
B~DRB1	B*08:01:01:01~DRB1*03:01:01:01SG	22	0.155	21	0.046	3.78	1.91–7.48	1.25E−05		Risk
DQA1~DQB1~DRB1~DRB345	DQA1*05:01:01:02~DQB1*02:01:01~ DRB1*03:01:01:01SG~DRB3*01:01:02:01/02	22	0.155	23	0.051	3.44	1.75–6.68	4.05E−05		Risk
DRB1~DRB345	DRB1*03:01:01:01SG~DRB3*01:01:02:01/02	22	0.155	23	0.051	3.44	1.75–6.68	4.05E−05		Risk
B	B*08:01:01:01	26	0.183	29	0.064	3.28	1.78–6.02	1.83E−05		Risk
DQA1~DRB1	DQA1*05:01:01:02~DRB1*03:01:01:01SG	23	0.162	26	0.057	3.18	1.66–6.02	7.36E−05		Risk
DQA1~DQB1	DQA1*05:01:01:02~DQB1*02:01:01	23	0.162	26	0.057	3.18	1.66–6.02	7.36E−05		Risk
DQA1	DQA1*05:01:01:02	23	0.162	26	0.057	3.18	1.66–6.02	7.36E−05		Risk
C~B	C*07:01:01:01~B*08:01:01:01	25	0.176	29	0.064	3.13	1.68–5.77	4.81E−05		Risk
A~B	A*01:01:01:01~B*08:01:01:01	19	0.134	24	0.053	2.77	1.38–5.46	0.001		Risk
A~C~B	A*01:01:01:01~C*07:01:01:01~B*08:01:01:01	19	0.134	25	0.055	2.65	1.33–5.19	0.002		Risk
A~C	A*01:01:01:01~C*07:01:01:01	19	0.134	25	0.055	2.65	1.33–5.19	0.002		Risk
DQB1~DRB1	DQB1*02:01:01~DRB1*03:01:01:01SG	26	0.183	38	0.084	2.45	1.37–4.34	8.41E−04		Risk
DQB1	DQB1*02:01:01	26	0.183	38	0.084	2.45	1.37–4.34	8.41E−04		Risk
DRB1	DRB1*03:01:01:01SG	26	0.183	38	0.084	2.45	1.37–4.34	8.41E−04		Risk
C	C*07:01:01:01	42	0.296	68	0.150	2.38	1.49–3.79	9.08E−05	7.26E-04	Risk
DRB345	DRB3*01:01:02:01/02	32	0.225	54	0.119	2.15	1.28–3.58	0.002		Risk
DQA1~DRB1	DQA1*01:02:02~DRB1*16:01:01	16	0.113	22	0.048	2.49	1.18–5.13	0.006		Risk
DRB1	DRB1*16:01:01	16	0.113	22	0.048	2.49	1.18–5.13	0.006		Risk
DQA1~DQB1~DRB1~DRB345	DQA1*01:02:02~DQB1*05:02:01~ DRB1*16:01:01~DRB5*02:02	15	0.106	21	0.046	2.44	1.13–5.11	9.53E−03		Risk
DRB1~DRB345	DRB1*16:01:01~DRB5*02:02	15	0.106	21	0.046	2.44	1.13–5.11	9.53E−03		Risk
DQA1	DQA1*01:02:02	20	0.141	29	0.064	2.40	1.24–4.56	3.57E−03		Risk
DRB345	DRB5*02:02	18	0.127	26	0.057	2.39	1.19–4.69	0.006		Risk
DQB1~DRB1	DQB1*05:02:01~DRB1*16:01:01	15	0.106	22	0.048	2.32	1.08–4.83	0.014		Risk
DQB1	DQB1*05:02:01	20	0.141	32	0.070	2.16	1.13–4.05	9.51E−03		Risk
DQA1~DQB1	DQA1*01:02:02~DQB1*05:02:01	18	0.127	29	0.064	2.13	1.07–4.11	0.015		Risk
A~C~B	A*02:01:01:01~C*07:01:01:01~B*18:01:01:02	11	0.077	11	0.024	3.38	1.29–8.80	0.003		Risk
A~B	A*02:01:01:01~B*18:01:01:02	11	0.077	15	0.033	2.46	0.99–5.88	0.024		Risk
A~C	A*02:01:01:01~C*07:01:01:01	15	0.106	24	0.053	2.12	1.00–4.34	0.026		Risk
										
DQB1	DQB1*02:02:01:01	4	0.028	35	0.077	0.35	0.09–1.00	0.040		Protective
DQA1~DRB1	DQA1*02:01:01:01SG~DRB1*07:01:01:01SG	4	0.028	38	0.084	0.32	0.08–0.91	0.024		Protective
DRB1	DRB1*07:01:01:01SG	4	0.028	38	0.084	0.32	0.08–0.91	0.024		Protective
DQA1	DQA1*02:01:01:01SG	4	0.028	39	0.086	0.31	0.08–0.88	0.020		Protective
DQA1~DQB1	DQA1*02:01:01:01SG~DQB1*02:02:01:01	3	0.021	33	0.073	0.28	0.05–0.90	0.024		Protective
DQB1~DRB1	DQB1*02:02:01:01~DRB1*07:01:01:01SG	3	0.021	32	0.070	0.28	0.06–0.93	0.029		Protective
DQB1~DRB1	DQB1*06:03:01~DRB1*13:01:01:01SG	2	0.014	27	0.059	0.23	0.03–0.92	0.028		Protective
DQA1~DQB1	DQA1*01:03:01:02SG~DQB1*06:03:01	2	0.014	27	0.059	0.23	0.03–0.92	0.028		Protective

EOMG, early-onset Myasthenia Gravis; Fq, frequency; OR, odds ratio; CI, confidence interval; P_adj, Bonferroni corrected P-values; Effect, effect of allele/haplotype on disease outcome.

**Table 3:** Significant associations of HLA alleles and haplotypes in Norwegian Myasthenia Gravis EOMG AChR-antibody positive and controls ranked by odds ratios.

HLA Locus	HLA alleles/haplotypes	EOMG Cases		Controls		OR	95% CI	*P*-value	Effect
		Count	Fq	Count	Fq				
B~DRB1	B*08:01:01:01~DRB1*03:01:01:01SG	78	0.302	105	0.108	3.57	2.51–5.05	7.24E−15	Risk
B	B*08:01:01:01	91	0.353	130	0.134	3.52	2.53–4.88	4.41E−16	Risk
B~C	B*08:01:01:01~C*07:01:01:01	85	0.329	127	0.131	3.26	2.33–4.54	6.43E−14	Risk
All	A*01:01:01:01~B*08:01:01:01~C*07:01:01:01~ DPA1*01:03:01:02~DPB1*04:01:01:01~ DQA1*05:01:01:02~DQB1*02:01:01~ DRB1*03:01:01:01SG~DRB3*01:01:02:01/02	25	0.097	31	0.032	3.25	1.80–5.80	8.84E−06	Risk
A~B	A*01:01:01:01~B*08:01:01:01	69	0.267	99	0.102	3.21	2.23–4.59	6.41E−12	Risk
A~C	A*01:01:01:01~C*07:01:01:01	67	0.260	99	0.102	3.09	2.14–4.42	4.66E−11	Risk
DRB1~DRB345	DRB1*03:01:01:01SG~DRB3*01:01:02:01/02	81	0.314	126	0.130	3.07	2.19–4.28	2.24E−12	Risk
DQA1~DQB1~DRB1~DRB345	DQA1*05:01:01:02~DQB1*02:01:01~ DRB1*03:01:01:01SG~DRB3*01:01:02:01/02	80	0.310	125	0.129	3.04	2.16–4.25	4.01E−12	Risk
DQA1~DRB1	DQA1*05:01:01:02~DRB1*03:01:01:01SG	80	0.310	125	0.129	3.04	2.16–4.25	4.01E−12	Risk
DQA1~DQB1	DQA1*05:01:01:02~DQB1*02:01:01	80	0.310	125	0.129	3.04	2.16–4.25	4.01E−12	Risk
DQA1	DQA1*05:01:01:02	80	0.310	125	0.129	3.04	2.16–4.25	4.01E−12	Risk
A~B~C	A*01:01:01:01~B*08:01:01:01~C*07:01:01:01	66	0.256	99	0.102	3.02	2.10–4.34	1.23E−10	Risk
DQB1~DRB1	DQB1*02:01:01~DRB1*03:01:01:01SG	82	0.318	131	0.135	2.98	2.13–4.15	5.53E−12	Risk
DRB1	DRB1*03:01:01:01SG	82	0.318	132	0.136	2.96	2.12–4.12	7.94E−12	Risk
DQB1	DQB1*02:01:01	82	0.318	132	0.136	2.96	2.12–4.12	7.94E−12	Risk
C	C*07:01:01:01	88	0.341	150	0.155	2.83	2.04–3.90	1.66E−11	Risk
DRB345	DRB3*01:01:02:01/02	86	0.333	161	0.166	2.51	1.82–3.46	2.52E−09	Risk
A	A*01:01:01:01	79	0.306	154	0.159	2.34	1.68–3.24	7.96E−08	Risk
B~DRB1	B*40:01:02~DRB1*13:02:01	15	0.058	23	0.024	2.54	1.21–5.17	0.005	Risk
DQA1~DRB1	DQA1*01:02:01:04SG~DRB1*13:02:01	23	0.089	40	0.041	2.28	1.27–3.98	1.93E−03	Risk
DQA1	DQA1*01:02:01:04SG	23	0.089	41	0.042	2.22	1.24–3.87	0.003	Risk
DRB1~DRB345	DRB1*13:02:01~DRB3*03:01:01	23	0.089	42	0.043	2.16	1.21–3.76	3.46E−03	Risk
DRB1	DRB1*13:02:01	23	0.089	42	0.043	2.16	1.21–3.76	0.003	Risk
DRB345	DRB3*03:01:01	23	0.089	42	0.043	2.16	1.21–3.76	0.003	Risk
DQA1~DQB1~DRB1~DRB345	DQA1*01:02:01:04SG~DQB1*06:04:01~ DRB1*13:02:01~DRB3*03:01:01	20	0.078	37	0.038	2.12	1.14–3.82	7.55E−03	Risk
DQA1~DQB1	DQA1*01:02:01:04SG~DQB1*06:04:01	20	0.078	37	0.038	2.12	1.14–3.82	7.55E−03	Risk
DQB1~DRB1	DQB1*06:04:01~DRB1*13:02:01	20	0.078	38	0.039	2.06	1.11–3.71	0.010	Risk
DQB1	DQB1*06:04:01	20	0.078	38	0.039	2.06	1.11–3.71	0.010	Risk
B~C	B*40:01:02~C*03:04:01:01	34	0.132	77	0.079	1.76	1.11–2.75	0.009	Risk
A	A*02:01:01:01	61	0.236	315	0.325	0.64	0.46–0.89	0.006	Protective
B~C	B*44:02:01:01~C*05:01:01:02	10	0.039	86	0.089	0.41	0.19–0.82	0.008	Protective
B	B*44:02:01:01	10	0.039	87	0.090	0.41	0.19–0.81	0.007	Protective
C	C*05:01:01:02	10	0.039	91	0.094	0.39	0.18–0.76	0.004	Protective
B~C	B*07:02:01~C*07:02:01:03	22	0.085	146	0.151	0.53	0.31–0.85	0.007	Protective
B	B*07:02:01	22	0.085	147	0.152	0.52	0.31–0.84	0.006	Protective
C	C*07:02:01:03	22	0.085	150	0.155	0.51	0.30–0.82	0.004	Protective
A~B~C	A*03:01:01:01~B*07:02:01~C*07:02:01:03	8	0.031	73	0.075	0.39	0.16–0.83	0.011	Protective
A~C	A*03:01:01:01~C*07:02:01:03	8	0.031	76	0.078	0.38	0.15–0.79	0.007	Protective
A~B	A*03:01:01:01~B*07:02:01	8	0.031	75	0.077	0.38	0.16–0.81	0.008	Protective
DQA1	DQA1*02:01:01:01SG	10	0.039	86	0.089	0.41	0.19–0.82	0.008	Protective
DQA1~DRB1	DQA1*02:01:01:01SG~DRB1*07:01:01:01SG	9	0.035	86	0.089	0.37	0.16–0.75	4.06E−03	Protective
DRB1	DRB1*07:01:01:01SG	9	0.035	86	0.089	0.37	0.16–0.75	0.004	Protective
DRB345	DRB3*02:02:01:02	7	0.027	71	0.073	0.35	0.14–0.78	0.007	Protective
DQB1	DQB1*06:03:01	5	0.019	64	0.066	0.28	0.09–0.70	0.004	Protective
DQA1~DRB1	DQA1*01:03:01:02SG~DRB1*13:01:01:01SG	4	0.016	64	0.066	0.22	0.06–0.61	1.63E−03	Protective
DQB1~DRB1	DQB1*06:03:01~DRB1*13:01:01:01SG	4	0.016	64	0.066	0.22	0.06–0.61	0.002	Protective
DQA1~DQB1	DQA1*01:03:01:02SG~DQB1*06:03:01	4	0.016	64	0.066	0.22	0.06–0.61	1.63E−03	Protective
DRB1	DRB1*13:01:01:01SG	4	0.016	64	0.066	0.22	0.06–0.61	0.002	Protective
DQA1	DQA1*01:03:01:02SG	4	0.016	65	0.067	0.22	0.06–0.60	0.001	Protective
DQA1~DQB1~DRB1~DRB345	DQA1*01:03:01:02SG~DQB1*06:03:01~ DRB1*13:01:01:01SG~DRB3*01:01:02:01/02	1	0.004	33	0.034	0.11	0.00–0.65	8.72E−03	Protective
DQB1	DQB1*03:03:02:01	1	0.004	34	0.035	0.11	0.00–0.65	0.007	Protective
DQA1~DQB1~DRB1~DRB345	DQA1*02:01:01:01SG~DQB1*03:03:02:01~ DRB1*07:01:01:01SG~DRB4*01:03:01:02N	1	0.004	34	0.035	0.11	0.00–0.65	7.48E−03	Protective
DQB1~DRB1	DQB1*03:03:02:01~DRB1*07:01:01:01SG	1	0.004	34	0.035	0.11	0.00–0.65	0.007	Protective
DQA1~DQB1	DQA1*02:01:01:01SG~DQB1*03:03:02:01	1	0.004	34	0.035	0.11	0.00–0.65	7.48E−03	Protective

EOMG, early-onset Myasthenia Gravis; Fq, frequency; OR, odds ratio; CI, confidence interval; *, statistically significant P-values; P-values were not corrected for multiple-comparisons; Effect, effect of haplotype on disease outcome.

**Table 4:** Significant associations of HLA alleles and haplotypes in Swedish EOMG AChR-antibody positive cases and controls ranked by odds ratios.

HLA Locus	HLA alleles/haplotypes	EOMG Cases		Controls		OR	95% CI	*P*-value	*P*_adj	Effect
		Count	Fq	Count	Fq					
B~C	B*08:01~C*07:01	75	0.341	456	0.108	4.29	3.15–5.81	<2.22e−16		Risk
B	B*08:01	76	0.345	469	0.111	4.24	3.12–5.74	<2.22e−16		Risk
B~DRB1	B*08:01~DRB1*03:01	62	0.282	387	0.091	3.91	2.81–5.38	<2.22e−16		Risk
A~B~C	A*01:01~B*08:01~C*07:01	55	0.250	347	0.082	3.74	2.65–5.21	<2.22e−16		Risk
A~B	A*01:01~B*08:01	55	0.250	350	0.083	3.70	2.63–5.16	<2.22e−16		Risk
A~C	A*01:01~C*07:01	56	0.255	375	0.088	3.52	2.50–4.89	4.29E−16		Risk
C	C*07:01	81	0.368	603	0.142	3.51	2.60–4.72	<2.22e−16		Risk
DQB1~DRB1	DQB1*02:01G~DRB1*03:01	65	0.295	519	0.122	3.01	2.18–4.10	1.18E−13		Risk
DRB1	DRB1*03:01	65	0.295	522	0.123	2.99	2.17–4.08	1.68E−13		Risk
A	A*01:01	63	0.286	596	0.141	2.45	1.78–3.35	2.82E−09		Risk
All	A*01:01~B*08:01~C*07:01~DPB1*04:01~DQB1*02:01G~DRB1*03:01	18	0.082	151	0.036	2.41	1.36–4.04	4.66E−04		Risk
DQB1	DQB1*02:01G	68	0.309	757	0.179	2.06	1.51–2.79	1.16E−06		Risk
DPB1	DPB1*01:01	23	0.105	234	0.055	2.00	1.21–3.16	2.19E−03	0.013	Risk
DPB1	DPB1*04:01	84	0.382	1909	0.450	0.75	0.56–1.00	0.047	0.279	NS
C	C*05:01	10	0.045	353	0.083	0.52	0.25–1.00	0.046		Protective
DQB1	DQB1*05:01	14	0.064	511	0.121	0.50	0.26–0.86	0.011		Protective
DQB1~DRB1	DQB1*05:01~DRB1*01:01	10	0.045	407	0.096	0.45	0.21–0.85	0.012		Protective
DRB1	DRB1*01:01	10	0.045	411	0.097	0.44	0.21–0.84	0.011		Protective
DRB1	DRB1*07:01	6	0.027	333	0.079	0.33	0.12–0.74	5.15E−03		Protective
DQB1~DRB1	DQB1*02:01~DRB1*07:01	3	0.014	231	0.054	0.24	0.05–0.72	8.07E−03		Protective

EOMG, early-onset Myasthenia Gravis; Fq, frequency; OR, odds ratio; CI, confidence interval; Effect, effect of haplotype on disease outcome. NS, not significant.

To further investigate the relationship between AChR-EOMG and the AH8.1, we performed allele-level association analyses in each of the three study populations. In the Italian and Norwegian cohorts, HLA-B*08:01:01:01 and HLA-DQA1*05:01:01:02 were the top two alleles strongly associated with AChR-EOMG risk (**Tables 2** and **3**). HLA-B*08:01:01:01 was the dominant risk allele (Italians, OR = 3.28, CI = 1.78–6.02, *P* = 1.83E−05; Norwegians, OR = 3.52, CI = 2.53–4.88, *P* = 4.41E−16).

**Figure 1:**
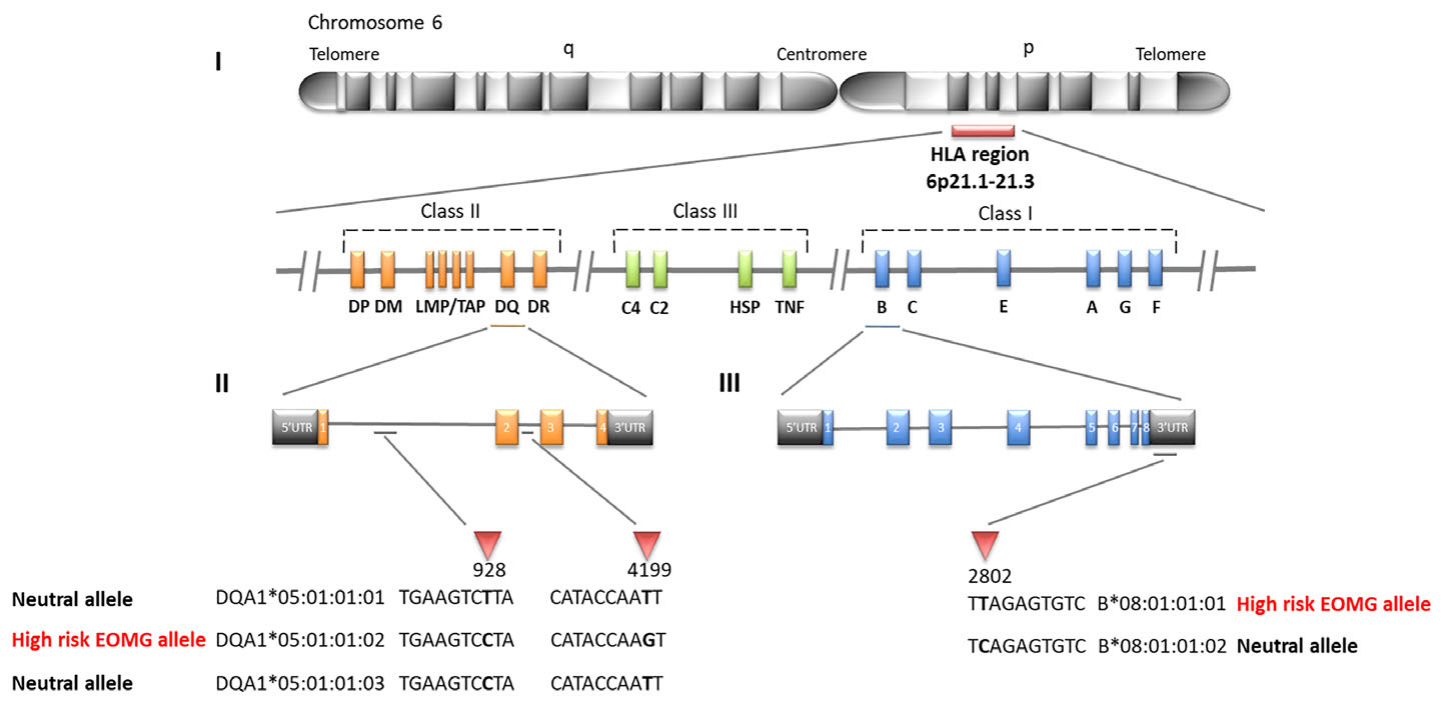
SNP differences in HLA alleles strongly associated with early-onset myasthenia gravis (EOMG) (I) Simplified map of the HLA region (II) Three different 4-field intronic variants of HLA-DQA1*05:01:01, only HLA-DQA1*05:01:01:02 is associated with high risk of EOMG the other alleles have neutral effects (III) Two 4-field variants of HLA-B*08:01:01 are distinguished by a SNP located in the 3’UTR; HLA-B*08:01:01:01 is a high risk EOMG allele whereas HLA-B*08:01:01:02 is neutral.

Likewise HLA-B*08:01 was the strongest risk factor in the Swedish cohort (**Table 4**; OR = 4.24, CI = 3.12–5.74, *P <*2.22E−16), followed by HLA-C*07:01 (OR = 3.51, CI = 2.60–4.72, *P <*2.22E−16); *HLA-DQA1* loci were not typed in Swedes. Analyses of alleles reduced to 2-field resolution in the Italian and Norwegian cohorts revealed a similar pattern of significant allele frequency differences between cases and controls (data not shown). In the Italian and Norwegian cohorts it is noteworthy that the effect size of the HLA-DQA1*05:01 allele at the protein level is considerably less than observed at the intronic level: Italians OR = 2.45, CI = 1.37–4.34, *P* = 8.41E−04; Norwegians OR = 2.96, CI = 2.12–4.12, *P* = 7.94E−12. The alternative 4-field variants HLA-DQA1*05:01:01:01 and HLA-DQA1*05:01:01:03 allele frequencies are considerably less than HLA-DQA1*05:01:01:02 in these populations. HLA-DQA1*05:01:01:01 and HLA-DQA1*05:01:01:03 are not present on the 8.1 ancestral haplotype. Furthermore allele HLA-DRB3*02:02:01:01 is a marker of HLA-DQA1*05:01:01:01 and HLA-DQA1*05:01:01:03 haplotypes, whereas HLA-DRB3*01:01:02:01/02 is found on the HLA-DQA1*05:01:01:02 haplotype, indicating that the HLA-DQA1*05:01:01 4-field haplotypes are distinct. The association results of HLA-DQA1*05:01 compared to HLA-DQA1*05:01:01:02 clearly suggest that the SNP variant located in intron 2 of the HLA-DQA1*05:01:01:02 gene is an important determinant for AChR-EOMG risk (**Figure 1**).

**Figure 2:**
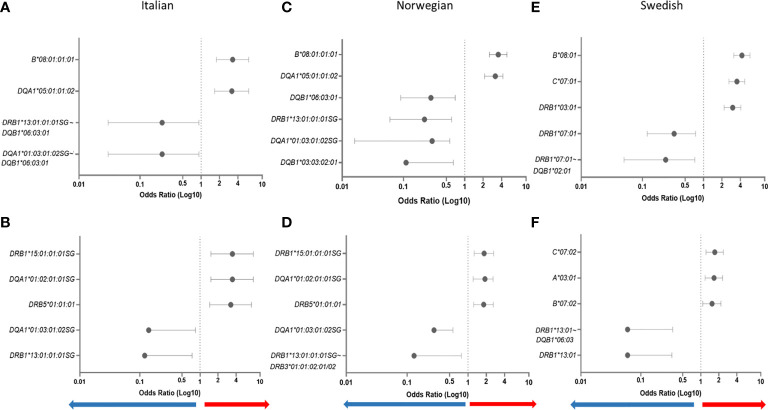
Forest plots depicting odds ratios and 95% confidence intervals of the most strongly associated HLA alleles with MG. HLA alleles/haplotypes in Italians EOMG **(A)** and LOMG **(B)**; Norwegians EOMG **(C)** and LOMG **(D)**; Swedish EOMG **(E)** and LOMG **(F)**.

In addition to the susceptibility factors described above we found that *HLA-DR16* alleles and haplotype-blocks (OR = 2.49, CI = 1.18–5.13, *P* = 0.006), and the HLA-A*02:01:01:01~HLA-C*07:01:01:01~HLA-B*18:01:01:02 haplotype (OR = 3.38, CI = 1.29–8.80, *P* = 0.003) were significantly associated with risk in Italians (**Table 2**). In Norwegians, HLA-DRB*13:02 class I and II haplotype blocks were also strong risk factors; HLA-B*40:01:02~HLA-DRB1*13:02:01, OR = 2.54, CI = 1.21–5.17, *P* = 0.005. Forest plots depicting the strongest statistically significant associated alleles with MG across groups are shown in **Figure 2**.

To determine whether sex influenced the associations observed we performed both case-control analyses stratified by sex and logistic regression adjusting for sex in Italian and Norwegian cohorts; sex data was not available for Swedish controls. Both sets of analyses for Italians and Norwegians produced very similar results to the original/unadjusted results (data not shown). Since the majority of AChR-EOMG cases in each three cohorts are comprised of females the association signals could be attributed to females. In logistic regression male sex had a negative coefficient estimate of ~−1.5 and the *P*-values were statistically significant indicating a negative correlation between males and AChR-EOMG.

We performed stratification analyses in the absence of the primary allele(s) with the strongest risk effects in order to identify secondary alleles associated with AChR-EOMG. Case-control analyses were performed in the absence of HLA-B*08:01:01:01, HLA-DQA1*05:01:01:02, and HLA-DRB1*03:01:01:01 (data not shown). In Norwegians when HLA-DQA1*05:01:01:02 was absent HLA-B*08:01:01:01 remained the strongest risk factor (OR = 3.72, CI = 1.69–7.78, *P*-adj = 6.40E−04). Removal of HLA-B*08:01:01:01 did not yield any significant associations after adjustment for multiple testing. In the Italian cohort, alleles of the *HLA-DR16* class II region (HLA-DRB1*16:01:01~HLA-DRB5*02:02~HLA-DQA1*01:02:02~HLA-DQB1*05:02:01) had a strong effect on AChR-EOMG risk (OR ~2.0), however, only HLA-DRB1*16:01:01 (average OR = 2.85, *P*-adj <0.036) and HLA-DQA1*01:02:02 (average OR = 2.58, *P*-adj <0.044) in the absence of HLA-B*08:01:01:01 and HLA-DQA1*05:01:01:02 were statistically significant when adjusted for multiple alleles. In Swedes, no significant associations were observed following correction for multiple comparisons.

### HLA-DRB1*13.1 and HLA-DRB1*07 Haplotypes Are Protective Against AChR-EOMG

Highly protective haplotype blocks in Italians include and HLA-DRB1*13:01:01:01SG~HLA-DQB1*06:03:01 and HLA-DQA1*01:03:01:02~HLA-DQB1*06:03:01 (OR = 0.23, CI = 0.03–0.92, *P* = 0.028). These haplotype blocks were also highly protective in AChR-EOMG Norwegian patients (OR = 0.22, CI = 0.06–0.61, *P* = 0.002 and 1.63E−03) as well as HLA-DRB1*13:01:01:01~HLA-DQA1*01:03:01:02, and HLA-DRB3*01:01:02:01/02~HLA-DRB1*13:01:01:01SG~HLA-DQA1*01:03:01:02~HLA-DQB1*06:03:01 which was strongly protective (OR = 0.11, CI = 0.00–0.65, *P* = 7.48E−03). Collectively these haplotype blocks comprise the extended class II HLA-DRB1*13:01:01:01SG haplotype (abbreviated here as HLA-DRB1*13.1). HLA-DRB*13.1 has been shown to confer protection for several autoimmune diseases such as rheumatoid arthritis and systemic lupus erythematosus, although the negative association with MG is not well established (37). However, it is noteworthy that *HLA-DR7* haplotype-blocks were also strongly protective in Norwegians, with OR values similar to those seen for the HLA-DRB1*13 haplotype-blocks. Specifically, haplotype-blocks which lie on the non-expressed HLA-DRB1*07:01:01:01SG~HLA-DRB4*01:03:01:02N~HLA-DQB1*03:03:02:01 were protective, whereas the expressed *HLA-DR7*~*HLA-DRB4* were not associated with protection. *HLA-DR7* was also found to be associated with protection in Italians but unlike Norwegians the expressed *HLA-DRB4* haplotype, as deduced from the *HLA-DQB1* allele, was protective.

Surprisingly, the HLA-DRB1*13.1 haplotype was not associated with AChR-EOMG in the Swedish cohort. Instead the HLA*DRB1*07:01~HLA-DQB1*02:01 haplotype was strongly protective (OR = 0.24, CI = 0.05–0.72, *P* = 8.07E−03), and the HLA-DRB1*01:01~HLA-DQB1*05:01 was moderately protective. At the allele-level only HLA-DRB1*07:01 had the strongest protective effect (OR = 0.33, CI = 0.12–0.74, *P* = 5.15E−03).

### Locus-Level Heterogeneity in AChR-LOMG Patients

Locus-level heterogeneity results for the AChR-LOMG subtype for three cohorts are shown in **Supplementary Table 3**. In the Italian cohort *HLA-DRB1* remained significant after adjustment for multiple comparisons. For the Norwegian group *HLA-DRB1*, *HLA-DQA1*, *HLA-DQB1*, *HLA-DRB1*~*HLA-DQB1*, and *HLA-DPA1*~*HLA-DPB1* were significant. In the Swedish group *HLA-DRB1*, *HLA-DQB1*, *HLA-C~HLA-B*, and *HLA-DRB1~HLA-DQB1* were significant.

### HLA-DRB*15.1 and HLA-DRB1*07 Are Risk Factors for AChR-LOMG Patients

In all three cohorts’ alleles and haplotype blocks of the *HLA-DR15~HLA-DR51~HLA-DQA1~HLA-DQB1* extended haplotype were positively associated with AChR-LOMG (**Tables 5**–**7**). However the effect sizes were significantly greater in the Italians with OR’s ranging from 3.26 to 3.56, compared to Norwegians; ORs ranging from 1.57 for HLA-C*07:02:01:03 to 3.23 for the extended haplotype encompassing both class I and II regions, and Swedes; OR = 1.54 for HLA-B*07:02 and largest OR of 2.30 for the extended haplotype.

**Table 5:** Significant associations of HLA alleles and haplotypes in Italian LOMG AChR-antibody positive cases and controls ranked by odds ratios.

HLA Locus	HLA alleles/haplotypes	LOMG Cases		Controls		OR	95% CI	*P*-value	*P*_adj	Effect
		Count	Fq	Count	Fq					
DQA1~DRB1	DQA1*01:02:01:01SG~DRB1*15:01:01:01SG	12	0.102	14	0.031	3.56	1.45–8.54	9.94E−04		Risk
DRB1~DRB345	DRB1*15:01:01:01SG~DRB5*01:01:01	14	0.119	17	0.037	3.46	1.52–7.71	5.19E−04		Risk
DRB1	DRB1*15:01:01:01SG	14	0.119	17	0.037	3.46	1.52–7.71	5.19E−04	4.67E−03	Risk
DQA1	DQA1*01:02:01:01SG	14	0.119	17	0.037	3.46	1.52–7.71	5.19E−04		Risk
DRB345	DRB5*01:01:01	14	0.119	18	0.040	3.26	1.45–7.18	8.79E−04		Risk
DPA1~DPB1	DPA1*01:03:01:02~DPB1*02:01:02	10	0.085	17	0.037	2.38	0.94–5.68	0.031		Risk
DQB1~DRB1	DQB1*02:02:01:01~DRB1*07:01:01:01SG	16	0.136	32	0.070	2.07	1.02–4.05	0.023		Risk
DQB1	DQB1*02:02:01:01	17	0.144	35	0.077	2.01	1.01–3.86	0.024		Risk
DQA1~DQB1	DQA1*02:01:01:01SG~DQB1*02:02:01:01	16	0.136	33	0.073	2.00	0.99–3.91	0.030		Risk
DQA1~DRB1	DQA1*02:01:01:01SG~DRB1*07:01:01:01SG	17	0.144	38	0.084	1.84	0.93–3.50	0.048		Risk
DRB1	DRB1*07:01:01:01SG	17	0.144	38	0.084	1.84	0.93–3.50	0.048		Risk
DRB345	DRB3*01:01:02:01/02	5	0.042	54	0.119	0.33	0.10–0.84	0.015		Protective
DQA1~DRB1	DQA1*01:03:01:02SG~DRB1*13:01:01:01SG	1	0.008	27	0.059	0.14	0.00–0.84	0.022		Protective
DQB1~DRB1	DQB1*06:03:01~DRB1*13:01:01:01SG	1	0.008	27	0.059	0.14	0.00–0.84	0.022		Protective
DQA1~DQB1	DQA1*01:03:01:02SG~DQB1*06:03:01	1	0.008	27	0.059	0.14	0.00–0.84	0.022		Protective
DQA1	DQA1*01:03:01:02SG	1	0.008	27	0.059	0.14	0.00–0.84	0.022		Protective
DRB1	DRB1*13:01:01:01SG	1	0.008	30	0.066	0.12	0.00–0.74	0.014	0.124	NS
A	A*24:02:01:01	4	0.034	57	0.126	0.24	0.06–0.68	4.06E−03		Protective

LOMG, late-onset Myasthenia Gravis; Fq, frequency; OR, odds ratio; CI, confidence interval; P_adj, Bonferroni corrected P-values, Only HLA-DRB1 alleles were corrected; Effect, effect of haplotype on disease outcome. NS, not significant.

**Table 6:** Significant associations of HLA alleles and haplotypes in Norwegian LOMG AChR-antibody positive cases and controls ranked by odds ratios.

HLA Locus	HLA alleles/haplotypes	LOMG Cases		Controls		OR	95% CI	*P*-value	*P*_adj	Effect
		Count	Fq	Count	Fq					
All loci	A*03:01:01:01~B*07:02:01~C*07:02:01:03~DPA1*01:03:01:02~DPB1*04:01:01:01~DQA1*01:02:01:01SG~ DQB1*06:02:01~DRB1*15:01:01:01SG~DRB5*01:01:01	12	0.055	17	0.018	3.23	1.38–7.30	1.30E−03	0.004	Risk
B~DRB1	B*07:02:01~DRB1*15:01:01:01SG	37	0.168	90	0.093	1.98	1.27–3.04	1.08E−03	0.009	Risk
DQA1~DRB1	DQA1*01:02:01:01SG~DRB1*15:01:01:01SG	54	0.245	145	0.149	1.85	1.27–2.67	5.74E−04	0.007	Risk
DRB1~DRB345	DRB1*15:01:01:01SG~DRB5*01:01:01	54	0.245	146	0.151	1.84	1.26–2.64	6.74E−04	0.009	Risk
DRB1	DRB1*15:01:01:01SG	54	0.245	146	0.151	1.84	1.26–2.64	6.74E−04		Risk
DQA1	DQA1*01:02:01:01SG	55	0.250	150	0.155	1.82	1.26–2.62	7.20E−04		Risk
DRB345	DRB5*01:01:01	54	0.245	148	0.153	1.81	1.24–2.60	9.23E−04	0.008	Risk
DQ~DRB	DQA1*01:02:01:01SG~DQB1*06:02:01~DRB1*15:01:01:01SG~ DRB5*01:01:01	52	0.236	144	0.148	1.78	1.21–2.57	1.50E−03	0.021	Risk
DQB1~DRB1	DQB1*06:02:01~DRB1*15:01:01:01SG	52	0.236	145	0.149	1.76	1.20–2.55	1.75E−03		Risk
DQA1~DQB1	DQA1*01:02:01:01SG~DQB1*06:02:01	52	0.236	145	0.149	1.76	1.20–2.55	1.75E−03		Risk
DQB1	DQB1*06:02:01	52	0.236	146	0.151	1.75	1.20–2.52	2.02E−03		Risk
B	B*07:02:01	50	0.227	147	0.152	1.65	1.12–2.39	6.36E−03	0.064	NS
B~C	B*07:02:01~C*07:02:01:03	49	0.223	146	0.151	1.62	1.10–2.35	8.99E−03	0.090	NS
C	C*07:02:01:03	49	0.223	150	0.155	1.57	1.07–2.27	0.015	0.160	NS
DPA1~DPB1	DPA1*01:03:01:04~DPB1*04:01:01:01	43	0.195	133	0.137	1.53	1.02–2.26	0.028	0.167	NS
DPA1	DPA1*01:03:01:04	49	0.223	154	0.159	1.52	1.03–2.20	0.023	0.159	NS
DQA1~DQB1	DQA1*02:01:01:01SG~DQB1*02:02:01:01	23	0.105	51	0.053	2.10	1.20–3.60	3.95E−03		Risk
DQB1~DRB1	DQB1*02:02:01:01~DRB1*07:01:01:01SG	23	0.105	51	0.053	2.10	1.20–3.60	3.95E−03		Risk
DQB1	DQB1*02:02:01:01	23	0.105	51	0.053	2.10	1.20–3.60	3.95E−03		Risk
DQ~DRB	DQA1*02:01:01:01SG~DQB1*02:02:01:01~ DRB1*07:01:01:01SG~DRB4*01:03:01:01/03	12	0.055	27	0.028	2.01	0.91–4.19	0.045	0.624	NS
DRB1~DRB345	DRB1*07:01:01:01SG~DRB4*01:03:01:01/03	12	0.055	27	0.028	2.01	0.91–4.19	0.045	0.579	NS
DQB1~DRB1	DQB1*02:01:01~DRB1*03:01:01:01SG	19	0.086	131	0.135	0.61	0.34–1.01	0.049		Protective
DQB1	DQB1*02:01:01	19	0.086	132	0.136	0.60	0.34–1.00	0.045		Protective
DRB1	DRB1*03:01:01:01SG	19	0.086	132	0.136	0.60	0.34–1.00	0.045		Protective
DRB345	DRB3*01:01:02:01/02	20	0.091	161	0.166	0.50	0.29–0.83	5.12E−03	0.046	Protective
DRB345	DRB3*02:02:01:02	7	0.032	71	0.073	0.42	0.16–0.92	0.025	0.226	NS
DQB1	DQB1*06:03:01	4	0.018	64	0.066	0.26	0.07–0.72	5.82E−03		Protective
DQA1~DRB1	DQA1*01:03:01:02SG~DRB1*13:01:01:01SG	3	0.014	64	0.066	0.20	0.04–0.61	2.36E−03	0.028	Protective
DRB1	DRB1*13:01:01:01SG	3	0.014	64	0.066	0.20	0.04–0.61	2.36E−03		Protective
DQB1~DRB1	DQB1*06:03:01~DRB1*13:01:01:01SG	3	0.014	64	0.066	0.20	0.04–0.61	2.36E−03		Protective
DQA1~DQB1	DQA1*01:03:01:02SG~DQB1*06:03:01	3	0.014	64	0.066	0.20	0.04–0.61	2.36E−03		Protective
DQA1	DQA1*01:03:01:02SG	3	0.014	65	0.067	0.19	0.04–0.60	2.08E−03		Protective
DQ~DRB	DQA1*01:03:01:02SG~DQB1*06:03:01~DRB1*13:01:01:01SG~ DRB3*01:01:02:01/02	1	0.005	33	0.034	0.13	0.00–0.78	0.018	0.250	NS
DRB1~DRB345	DRB1*13:01:01:01SG~DRB3*01:01:02:01/02	1	0.005	33	0.034	0.13	0.00–0.78	0.018	0.232	NS

LOMG, late-onset Myasthenia Gravis; Fq, frequency; OR, odds ratio; CI, confidence interval; P_adj, Bonferroni corrected P-values, P-values for HLA-DQA1, HLA-DQB1, HLA-DRB1, HLA-DQA1~HLA-DQB1, and HLA-DQB1~HLA-DRB1 alleles were not corrected for multiple comparisons; Effect, effect of haplotype on disease outcome. NS, not significant.

**Table 7:** Significant associations of HLA alleles and haplotypes in Swedish LOMG AChR-antibody positive cases and controls ranked by odds ratios.

HLA Locus	HLA alleles/haplotypes	LOMG Cases		Controls		OR	95% CI	*P*-value	*P*_adj	Effect
		Count	Fq	Count	Fq					
All	A*03:01~B*07:02~C*07:02~DPB1*04:01~DQB1*06:02~DRB1*15:01	15	0.068	131	0.031	2.30	1.23–4.02	2.44E−03	0.005	Risk
A~C	A*03:01~C*07:02	27	0.123	271	0.064	2.05	1.29–3.14	6.59E−04	0.005	Risk
A~B	A*03:01~B*07:02	24	0.109	262	0.062	1.86	1.14–2.91	5.23E−03	0.031	Risk
A~B~C	A*03:01~B*07:02~C*07:02	23	0.105	252	0.059	1.85	1.12–2.92	6.68E−03	0.053	NS
C	C*07:02	53	0.241	663	0.156	1.71	1.22–2.37	8.67E−04	0.009	Risk
B~C	B*07:02~C*07:02	46	0.209	583	0.138	1.66	1.16–2.34	2.93E−03		Risk
A	A*03:01	52	0.236	666	0.157	1.66	1.18–2.31	1.81E−03	0.016	Risk
B~DRB1	B*07:02~DRB1*15:01	30	0.136	387	0.091	1.57	1.02–2.36	0.025	0.151	NS
B	B*07:02	46	0.209	620	0.146	1.54	1.08–2.17	0.011	0.107	NS
DQB1~DRB1	DQB1*04:02~DRB1*08:01	15	0.068	154	0.036	1.94	1.04–3.38	0.016		Risk
DRB1	DRB1*08:01	15	0.068	154	0.036	1.94	1.04–3.38	0.016		Risk
B	B*35:01	18	0.082	218	0.051	1.64	0.94–2.73	0.050	0.495	NS
DQB1~DRB1	DQB1*02:01G~DRB1*07:01	19	0.086	231	0.054	1.64	0.95–2.69	0.045		Risk
B~C	B*40:01~C*03:04	25	0.114	324	0.076	1.55	0.96–2.40	0.045		Risk
DPB1	DPB1*02:01	39	0.177	552	0.130	1.44	0.98–2.07	0.045	0.268	NS
										
DQB1~DRB1	DQB1*02:01G~DRB1*03:01	17	0.077	519	0.122	0.60	0.34–1.00	0.045		Protective
DRB1	DRB1*03:01	17	0.077	522	0.123	0.60	0.34–0.99	0.042		Protective
DQB1	DQB1*03:01	20	0.091	654	0.154	0.55	0.33–0.88	0.011		Protective
B~C	B*44:02~C*05:01	7	0.032	296	0.070	0.44	0.17–0.93	0.029		Protective
C	C*05:01	8	0.036	353	0.083	0.42	0.18–0.84	0.013	0.129	NS
A~C	A*02:01~C*05:01	4	0.018	213	0.050	0.35	0.09–0.92	0.031	0.218	NS
A~B~C	A*02:01~B*44:02~C*05:01	3	0.014	189	0.045	0.30	0.06–0.89	0.027	0.220	NS
DQB1	DQB1*06:03	2	0.009	313	0.074	0.12	0.01–0.42	2.58E−04		Protective
DQB1~DRB1	DQB1*06:03~DRB1*13:01	1	0.005	300	0.071	0.06	0.00–0.34	1.35E−04		Protective
DRB1	DRB1*13:01	1	0.005	308	0.073	0.06	0.00–0.33	1.05E−04		Protective

LOMG, late-onset Myasthenia Gravis; Fq, frequency; OR, odds ratio; CI, confidence interval; P_adj, Bonferroni corrected P-values, P-values for HLA-B~C, HLA-DQB1~HLA-DRB1, HLA-DRB1, and HLA-DQB1 alleles were not corrected for multiple comparisons; Effect, effect of allele/haplotype on disease outcome. NS, not significant.

The association of HLA-DRB1*15:01 with LOMG risk has been previously reported (26). However, in this study we find that HLA-DQA1*01:02:01:01 has an equivalent strength of association as HLA-DRB1*15:01:01:01SG for AChR-LOMG risk in Italians (OR = 3.46, CI = 1.52–7.71, P = 5.19E−04) and in Norwegians very similar effect sizes were found (HLA-DRB1*15:01:01:01, OR = 1.84, CI = 1.26–2.64, *P* = 6.74E−04; HLA-DQA1*01:02:01:01, OR = 1.82, CI = 1.26–2.62, *P* = 7.20E−04). Furthermore, at the haplotypic-level in the Italian cohort, HLA-DQA1*01:02:01:01SG~HLA-DRB1*15:01:01:01SG (*D*’ = 0.89) was more strongly associated than the ‘fixed’ HLA-DRB1*15:01:01:01~HLA-DRB5*01:01:01 haplotype where *D*’ = 1. These results indicate that both alleles are the strongest determinants of AChR-LOMG risk. In Norwegians a similar pattern was observed (**Table 6**).

Also it is noteworthy that the HLA-DQA1*02:01:01:01SG~HLA-DQB1*02:02:01:01, which had the second largest effect size for risk outside of the HLA-DRB*15 haplotype in the Norwegian cohort (OR = 2.10, CI = 1.20–3.60, *P* = 3.95E−03) and a similar effect size in Italians (OR = 2.00, CI = 0.99–3.91, *P* = 0.030) forms part of the HLA-DRB1*07:01:01:01SG~HLA-DRB4*01 expressed haplotype block. These results suggest that the *HLA-DQ* region of the *HLA-DR7* haplotype is an additional risk factor for AChR-LOMG.

Restricting the dataset to individuals without alleles located on the *HLA-DR15* class II haplotype lends further support to the involvement of *HLA-DR7* alleles as secondary risk factors in AChR-LOMG. In the Norwegian group HLA-DQB1*02:02:01:01 (OR = 2.7, *P* = 5.0E−04) and HLA-DRB1*07:01:01:01SG (OR = 1.98, *P* = 8.4E−03) were significantly associated with susceptibility in the absence of both, HLA-DQA1*01:02:01:01SG and HLA-DRB1*15:01:01:01SG alleles (data not shown). Finally, in the stratification analyses of Italians these alleles were positively associated with AChR-LOMG but only the adjusted HLA-DQB1*02:02:01:01 P-value were significant (OR = 2.39, CI = 1.17 to 4.71, *P* = 0.046).

As found for the AChR-EOMG cohort, strongly protective alleles and haplotypes were observed at the HLA-DRB1*13:01:01:01SG class II region haplotype in all three populations. Somewhat surprisingly, the HLA-DRB1*03:01~HLA-DQB1*02:01 haplotype block, which was a susceptibility factor for AChR-EOMG, was found to be weakly protective for AChR-LOMG Swedish patients; OR = 0.60, CI = 0.34–1.00, *P* = 0.045.

### Generalized and Ocular MG Subsets

We examined whether the allele distribution varied with generalized and ocular subsets within the aforementioned EOMG and LOMG non-thymomatous AChR positive antibody datasets. In the generalized subset similar associations were observed as described above. For both EOMG and LOMG ocular tests, no significant associations were found following adjustment for multiple testing.

## Discussion

We provide a detailed MG association analyses data derived from high-depth sequencing of complete HLA genes (*HLA-A*, *HLA-C*, *HLA-B*, *HLA-DQA1*, *HLA-DPA1*) and wide coverage of genomic regions (*HLA-DRB1*, *HLA-DRB3/4/5*, *HLA-DQB1*, *HLA-DPB1*). In contrast to previous reports of HLA associations with MG, which have often utilized serological and low to mid-resolution molecular typing methods, we evaluated individuals from three European populations in a single study and utilized next-generation sequencing to capture variation in intronic and untranslated regions.

Our results have extended and refined previous observations of HLA alleles with MG risk. Consistent with previous HLA MG association studies conducted in European populations (20, 22, 26, 38), we showed that the ancestral haplotype 8.1 most common in Northern European populations is associated with AChR-EOMG risk in Italian, Norwegian, and Swedish cohorts. However, in the Italian and Norwegian cohorts, who were examined at maximum HLA allelic resolution, we successfully sequenced the intronic and UTR variants for most alleles composing the AH8.1 haplotype. This is clearly illustrated when we consider the dominant AChR-EOMG risk allele HLA-B*08:01 which according to the IMGT/HLA Database release 3.250 has five 3-field variants (alleles that differ by synonymous mutations and are translated into the same protein), and two 4-field variants named HLA-B*08:01:01:01 and HLA-B*08:01:01:02 that are distinguishable due to a single SNP T > C at 2,802 bp in the 3’ UTR of the HLA-B gene. In addition, we showed conclusively that the frequency of HLA-B*01:01:01:01 was over represented in Italian and Norwegian AChR-EOMG patients compared to controls; in fact HLA-B*08:01:01:02 occurs at much lower frequencies (AF ~0.005, n = 1 or 2) in the Italian group regardless of disease status, and was not observed at all in Norwegians. Although not shown, HLA-B*01:01:01:02 was not observed in the Swedish AChR-EOMG patient cohort therefore HLA-B*08:01:01:01 is also the risk allele in this group. Furthermore, we show that HLA-B*08:01:01:01 is almost exclusively linked to HLA-C*07:01:01:01 (*D*’ >0.95) of which there are five other 4-field variants that differ due to various SNPs located within introns. These observations also extend to the class II region of the AH8.1 haplotype; HLA-DQA1*05:01:01:02 was identified as the risk allele whereas HLA-DQA1*05:01:01:01 and HLA-DQA1*05:01:01:03 are neutral. Due to the presence of a prominent low-complexity sequence region near the 5’ end of intron 2 in the *HLA-DRB* loci and unsequenced regions it was not possible to define the 4-field variant of HLA-DRB1*03:01:01 and HLA-DRB3*01:01:02. We note that increased allelic resolution has decreased the statistical power of the association analyses by increasing the number of categories. We observed this event when we compare the 4-field association results to the 2-field data where overall the effect size is greater and *P*-values smaller. The downside is that lower resolution typing pools a large number of HLA alleles together and therefore masks the effects of individual yet pertinent alleles. One of the main take home messages from this study is that specific intronic and UTR variants of HLA alleles contribute to MG pathogenesis.

Since the AH8.1 haplotype is most frequent and highly conserved in Europeans it is not too surprising that alleles of this haplotype and extended regions have been found to be associated with several immune system dysfunctions including chronic inflammation and autoimmune diseases such as Type I diabetes (39) and systemic lupus erythematosus (40). Autoimmunity is thought, in part, to result from the downregulation of type II cytokine responses, which is influenced by the interaction of alleles on AH8.1, leading to enhanced humoral responses and increased production of autoantibodies (41). On the other hand, the stability of the AH8.1 haplotype, and relatively low recombination rate across the haplotype suggests that this haplotype is positively selected and has a survival advantage. The AH8.1 contributes to EOMG in European populations (26, 38, 42) but not populations from East Asia where HLA-DRB1*09:01 is the main risk allele for EOMG (43, 44). The numerous studies conducted in Europeans have shown that HLA-B*08:01 appears to be the dominant risk allele for EOMG and the other associated alleles located on AH8.1 are simply a consequence of strong LD between them and HLA*08:01. In this present study, this notion appears to be the case when we only consider the reduced 2-field data for all three groups; HLA-B*08:01 has the strongest effect. However, at the 4-field resolution HLA-DQA1*05:01:01:02 has a similar effect size to HLA-B*08:01:01:01 for risk in Italian patients. This finding is of interest as to the best of our knowledge the HLA-DQA1*05:01:01 allele has never been investigated in previous MG association studies.

We identified additional risk factors for EOMG that appeared to be ethnic specific such as the HLA-DRB1*16:01:01 class II haplotype-blocks in Italians, and the HLA-DRB1*13:02:01 haplotype-blocks encompassing class I and II alleles in Norwegians. These findings are novel and should be confirmed and further examined to refine the SNPs associated with EOMG in these European populations.

We also confirmed previous findings of a protective effect conferred by the HLA-DRB1*13:01:01:01SG class II allele and haplotype in both AChR-EOMG and AChR-LOMG patients from Italy, Norway, and Sweden. HLA-DRB1*13:01:01:01SG has a frequency of ~0.05 in several European populations (unpublished observations). The protective effect of the HLA-DRB1*13:01 allele is not exclusive to MG but has also been found to be negatively associated with a plethora of autoimmune diseases in Europeans, and Asians (37, 45). It has been hypothesized that the protective effect of HLA-DRB1*13:01 is due to the high binding affinity/avidity of T-cell receptors on autoreactive regulatory T-cells (Tregs) for HLA-DRB1*13:01 molecules. Consequently, thymic negative selection and autoreactive Tregs development are enhanced resulting in decreased pathogenicity (41).

A novel EOMG protective allele HLA-DRB1*07:01 was detected in all three cohorts. Specifically, the HLA-DRB1*07:01:01:01SG~HLA-DRB4*01:03:01:02N~HLA-DQA1*02:01:01:01SG~HLA-DQB1*03:03:02:01 class II extended haplotype and the 2-loci *HLA-DRB1~HLA-DQB1*, *HLA-DQA1-DQB1* blocks of this haplotype were equally strongly protective (OR = 0.11) in the Norwegian cohort. Due to strong LD across this region and similarity of effect sizes we cannot easily discern which allele is causative from the haplotype association analyses. HLA-DRB1*07:01:01:01SG of the expressed *HLA-DR7* haplotype also occurred at higher allele frequencies in Italian control subjects (AF = 0.084) compared to AChR-EOMG patients (AF = 0.028). HLA-DRB1*07:01 has been found to be associated with protection in systemic sclerosis in European origin individuals (46). The amino acid differences between the protective alleles HLA-DRB1*13:01 and HLA-DRB1*07:01 are substantial, therefore they are highly likely to present different repertoires of peptides suggesting that the immunological mechanisms underlying protection differs. Interestingly, we find that the HLA-DRB1*07:01:01:01SG negatively associated in EOMG has the opposite predisposing effect in LOMG, further underscoring the fact that they are distinct subtypes of MG with different immunogenetic backgrounds.

LOMG risk has been shown to be positively associated with the HLA-DRB1*15:01 allele in a Norwegian population (26), in this study we replicate and extrapolate those findings. Specifically, we find that the extended haplotype, HLA-A*03:01:01:01~HLA-C*07:02:01:03~ HLA-B*07:02:01~HLA-DRB1*15:01:01:01SG~HLA-DRB5*01:01:01~HLA-DQA1*01:02:01:01SG~HLA-DQB1*06:02:01~HLA-DPA1*01:03:01:02~HLA-DPB1*04:01:01:01 strongly predisposes to risk (OR = 3.23), in which the associations of the *HLA-DP* loci reflect LD with the susceptible *HLA-DR*-*HLA-DQ* haplotype. The effect of the extended haplotype was substantially greater than the alleles and the various two to three loci haplotype blocks associated with risk. These findings allude to a synergistic risk effect of the *HLA-DR15* alleles, although the independent effects of HLA-DRB1*15:01:01:01SG, HLA-DRB5*01:01:01, and HLA-DQA1*01:02:01:01 were the only alleles statistically significant after adjustment for multiple alleles. We observed a similar *HLA-DR15* distribution pattern in Italian AChR-LOMG patients, however the strongest haplotype associated was HLA-DRB1*15:01:01:01SG~HLA-DQA1*01:02:01:01SG (OR = 3.56). The sample size of the Norwegian AChR-LOMG cohort is larger than the Italian counterparts, thus this could account for the slight discrepancy in results. Interestingly, in Italians and Norwegians the strongest signal for susceptibility resided in the class II component of the *HLA-DR15* haplotype, whereas in Swedes with the exception of the extended haplotype the strongest effect was seen in the class I region of the *HLA-DR15* haplotype. These findings indicate that it is difficult to map the susceptible factor based on HLA alleles, and further studies are needed to identify the causative SNP(s).

In conclusion, this study provides additional evidence that specific HLA alleles and haplotypes play essential roles in the pathogenesis of different subtypes of MG. Distinctive HLA factors for susceptibility and resistance were found in EOMG and LOMG, and open avenues for investigating the mechanisms for these distinct diseases. The extensive LD within the HLA region requires that populations of non-European background should be investigated to distinguish between primary and secondary effects. Due to the location of many of the causative polymorphisms in non-coding regions that could affect expression functional assays of candidate genes are optimal to further understand MG pathogenesis. A deeper understanding of the biological mechanisms underlying MG and could offer new insights for diagnostic approaches and the development of novel therapies.

## Data Availability Statement

The datasets presented in this study can be found in online repositories. The names of the repository/repositories and accession number(s) can be found below: (www.immport.org/shared/home), ImmPort/.

## Author Contributions

LC was involved in the conception and design of the study, performed the statistical analyses, interpreted the results, and wrote the manuscript. MF-V and JO were involved in the conception and design of the study, contributed to the interpretation of the results, and contributed to the final version of the manuscript. JH contributed to the conception of the study and interpretation of the results. PC, RF, BL, HH, SB, JO, PB, AH, LH, and RM contributed to collection of samples and clinical data. SC was responsible for sample and data collection at the UCSF-DNA bank. LC and SG performed NGS genotyping assays. MB contributed genotyping data. All authors contributed to the article and approved the submitted version.

## Funding

This work was supported by grant U19NS095774 (JO and MF-V) from the U.S. National Institutes of Health (NIH). The UCSF DNA biorepository is supported by RG-1611-26299 from the National Multiple Sclerosis Society.

## Conflict of Interest

The authors declare that the research was conducted in the absence of any commercial or financial relationships that could be construed as a potential conflict of interest.

## Abbreviations

MG, myasthenia gravis; HLA, human leukocyte antigen; NGS, next-generation sequencing; EOMG, early onset myasthenia gravis; LOMG, late onset myasthenia gravis; AChR, acetylcholine receptor.
