# Supplementary material for: Next-Generation Sequencing Identifies Extended HLA Class I and II Haplotypes Associated With Early-Onset and Late-Onset Myasthenia Gravis in Italian, Norwegian, and Swedish Populations
Source: Front Immunol. 2021 Jun 7;12:667336. doi: 10.3389/fimmu.2021.667336 (PMC8215161; doi:10.3389/fimmu.2021.667336)
Supplement: Supplementary file 1 [file Table_1.doc]

| **Supplementary Table 1.** Characteristics of ambiguous HLA class II alleles | | | | | |
| --- | --- | --- | --- | --- | --- |
| **Ambiguity group** | **Alleles in ambiguity group** | **Reason for ambiguity** | **Gene region** | **Position** | **Motif** |
| HLA-DQA1*01:01:01:02SG | HLA-DQA1*01:01:01:02 | STR (mononucleotide) | Intron 1 | 3107 to 3118 bp | A |
|  | HLA-DQA1*01:01:01:03 |  |  |  |  |
| HLA-DQA1*01:02:01:01SG | HLA-DQA1*01:02:01:01 | STR (mononucleotide) | Intron 1 | 3100 to 3113 bp | A |
|  | HLA-DQA1*01:02:01:03 |  |  | 3355 to 3368 bp | A |
|  | HLA-DQA1*01:02:01:05 |  |  |  |  |
| HLA-DQA1*01:02:01:04SG | HLA-DQA1*01:02:01:04 | STR (mononucleotide) | Intron 1 | 3108 to 3121 bp | A |
|  | HLA-DQA1*01:02:01:06 |  |  |  |  |
|  | HLA-DQA1*01:02:01:07 |  |  |  |  |
| HLA-DQA1*01:03:01:02SG | HLA-DQA1*01:03:01:02 | STR (mononucleotide) | Intron 1 | 3105 to 3114 bp | A |
|  | HLA-DQA1*01:03:01:06 |  |  |  |  |
| HLA-DQA1*01:03:01:03SG | HLA-DQA1*01:03:01:03 | STR (mononucleotide) | Intron 1 | 3104 to 3118 bp | A |
|  | HLA-DQA1*01:03:01:04 |  |  | 3360 to 3374 bp | A |
| HLA-DQA1*01:04:01:01SG | HLA-DQA1*01:04:01:01 | STR (mononucleotide) | Intron 1 | 3353 to 3368 bp | A |
|  | HLA-DQA1*01:04:01:02 |  |  |  |  |
|  | HLA-DQA1*01:04:01:04 |  |  |  |  |
| HLA-DQA1*02:01:01:01SG | HLA-DQA1*02:01:01:01 | STR (mononucleotide) | Intron 3 | 4836 to 4848 bp | T |
|  | HLA-DQA1*02:01:01:02 |  |  |  |  |
| HLA-DQA1*05:05:01:01SG | HLA-DQA1*05:05:01:01 | STR (tetranucleotide) | Intron 3 | 4947 to ~5028 bp | TTTC |
|  | HLA-DQA1*05:05:01:02 |  |  |  |  |
| HLA-DQA1*05:05:01:05SG | HLA-DQA1*05:05:01:05 | STR (tetranucleotide) | Intron 3 | 4952 to 5033 bp | TTTC |
|  | HLA-DQA1*05:05:01:06 |  |  |  |  |
| HLA-DQB1*03:03:02:02 | HLA-DQB1*03:03:02:02 | Unsequenced region | Intron 5 | 6488 bp | T>G SNP |
|  | HLA-DQB1*03:03:02:03 |  |  |  |  |
| HLA-DQB1*05:03:01:01 | HLA-DQB1*05:03:01:01 | Unsequenced region | 5’UTR | -157 bp | C>T SNP |
|  | HLA-DQB1*05:03:01:02 |  |  |  |  |
|  |  |  |  |  |  |
|  |  |  |  |  |  |
|  |  |  |  |  |  |
|  |  |  |  |  |  |
| HLA-DRB1*03:01:01:01SG | HLA-DRB1*03:01:01:01 | Unsequenced region | Intron 1 | 1522 bp | A>T SNP |
|  | HLA-DRB1*03:01:01:02 |  |  |  |  |
|  |  | STR (dinucleotide) | Intron 2 | 8412 to ~8465 bp | GT |
|  |  |  |  | ~8466 to ~8477 bp | GA |
| HLA-DRB1*04:01:01:01SG | HLA-DRB1*04:01:01:01 | STR (dinucleotide) | Intron 2 | 8676 to ~8719 bp | GT |
|  | HLA-DRB1*04:01:01:02 |  |  | ~8720 to ~8749 bp | GA |
| HLA-DRB1*07:01:01:01SG | HLA-DRB1*07:01:01:01 | Unsequenced region | Intron 1 | 11734-35 bp | Indel CA |
|  | HLA-DRB1*07:01:01:02 |  |  | 7929 bp | G>A SNP |
|  |  | STR (trinucleotide) | Intron 5 | 14786 to 14890 bp | GAA |
|  |  |  |  |  |  |
| HLA-DRB1*13:01:01:01SG | HLA-DRB1*13:01:01:01 | STR (dinucleotide) | Intron 2 | ~8417 to ~8462 bp | GT |
|  | HLA-DRB1*13:01:01:02 |  |  | ~8463 to ~8504 bp | GA |
| HLA-DRB1*15:01:01:01SG | HLA-DRB1*15:01:01:01 | STR (dinucleotide) | Intron 2 | 5701 to 5740 bp | GT |
|  | HLA-DRB1*15:01:01:02 |  |  | 5741 to 5784 bp | CA |
|  | HLA-DRB1*15:01:01:03 |  |  |  |  |
| HLA-DRB1*15:02:01:01 | HLA-DRB1*15:02:01:01 | Unsequenced region | Intron 1 | 2146 bp | G>C SNP |
|  | HLA-DRB1*15:02:01:02 |  |  |  |  |
|  |  | STR (dinucleotide) | Intron 2 | 5685 to 5742 bp | GT |
| HLA-DRB1*15:03:01:01SG | HLA-DRB1*15:03:01:01 | STR (dinucleotide) | Intron 2 | 5682 to 5717 bp | GT |
|  | HLA-DRB1*15:03:01:02 |  |  |  |  |
| HLA-DRB3*01:01:02:01 | HLA-DRB3*01:01:02:01 | Unsequenced region | Intron sequence not available for | | |
|  | HLA-DRB3*01:01:02:02 |  | HLA-DRB3*01:01:02:02 | | |
| HLA-DRB4*01:03:01:01 | HLA-DRB4*01:03:01:01 | Unsequenced region | Intron 1 | 3616 bp | G>A SNP |
|  | HLA-DRB4*01:03:01:03 |  | Intron 1 | 7069 bp | C>A SNP |
| HLA-DPB1*02:01:02 | HLA-DPB1*02:01:02 | Unsequenced region | Exon 5 | codon 225 | CAA>CAG |
|  | HLA-DPB1*02:01:19 |  |  |  |  |
| HLA-DPB1*13:01:01 | HLA-DPB1*13:01:01 | Unsequenced region | Exon 1 | codon -22 | GCG>GCA |
|  | HLA-DPB1*107:01 |  |  | codon -14 | ACG>ATG |
|  |  |  |  |  |  |
