# Supplementary material for: Next-Generation Sequencing Identifies Extended HLA Class I and II Haplotypes Associated With Early-Onset and Late-Onset Myasthenia Gravis in Italian, Norwegian, and Swedish Populations
Source: Front Immunol. 2021 Jun 7;12:667336. doi: 10.3389/fimmu.2021.667336 (PMC8215161; doi:10.3389/fimmu.2021.667336)
Supplement: Supplementary file 2 [file Table_2.doc]

**Supplementary Table 2.** Locus-level heterogeneity between EOMG AChR-antibody positive cases and controls

|  | **Italian** | | | | **Norwegian** | | | | **Swedish** | | | |
| --- | --- | --- | --- | --- | --- | --- | --- | --- | --- | --- | --- | --- |
| HLA Locus | *χ*^2^ | d.f. | *P*_adj | Significance | *χ*^2^ | d.f. | *P*_adj | Significance | *χ*^2^ | d.f. | *P*_adj | Significance |
| A | 12.606 | 7 | 1.646 | NS | 49.736 | 9 | 2.54E-06 | * | 38.622 | 9 | 1.75E-04 | * |
| C | 19.085 | 8 | 0.288 | NS | 69.438 | 12 | 8.57E-09 | * | 92.399 | 10 | 2.33E-14 | * |
| B | 24.507 | 7 | 0.019 | * | 86.435 | 11 | 1.74E-12 | * | 118.568 | 10 | 2.89E-15 | * |
| DRB345 | 28.819 | 8 | 0.007 | * | 59.386 | 10 | 9.95E-08 | * | N/T | N/T | N/T | N/T |
| DRB1 | 29.749 | 8 | 0.005 | * | 77.739 | 11 | 8.47E-11 | * | 68.801 | 10 | 9.82E-10 | * |
| DQA1 | 39.463 | 10 | 4.21E-04 | * | 78.162 | 11 | 7.02E-11 | * | N/T | N/T | N/T | N/T |
| DQB1 | 31.076 | 8 | 0.003 | * | 75.367 | 11 | 2.42E-10 | * | 35.118 | 9 | 7.38E-04 | * |
| DPA1 | 11.101 | 8 | 3.921 | NS | 6.613 | 8 | 12.156 | NS | N/T | N/T | N/T | N/T |
| DPB1 | 4.868 | 7 | 13.522 | NS | 3.992 | 6 | 14.232 | NS | 12.649 | 6 | 0.637 | NS |
| A~C | 15.860 | 2 | 0.007 | * | 63.865 | 11 | 3.70E-08 | * | 72.133 | 8 | 2.40E-11 | * |
| A~B | 16.714 | 3 | 0.016 | * | 71.752 | 9 | 1.45E-10 | * | 73.495 | 7 | 3.77E-12 | * |
| C~B | 22.714 | 5 | 0.008 | * | 85.622 | 11 | 2.51E-12 | * | 115.476 | 8 | 2.89E-15 | * |
| A~C~B | 19.507 | 2 | 0.001 | * | 65.277 | 8 | 8.93E-10 | * | 74.843 | 8 | 6.90E-12 | * |
| B~DRB1 | 20.092 | 2 | 0.001 | * | 78.939 | 9 | 5.51E-12 | * | 89.353 | 6 | 2.89E-15 | * |
| DRB345~DRB1 | 32.629 | 8 | 0.001 | * | 80.859 | 14 | 4.12E-10 | * | N/T | N/T | N/T | N/T |
| DRB345~DRB1~DQA1~DQB1 | 33.429 | 8 | 0.001 | * | 85.040 | 15 | 1.74E-10 | * | N/T | N/T | N/T | N/T |
| DRB1~DQA1 | 34.130 | 8 | 0.001 | * | 82.599 | 12 | 2.76E-11 | * | N/T | N/T | N/T | N/T |
| DRB1~DQB1 | 30.766 | 8 | 0.003 | * | 81.158 | 13 | 1.40E-10 | * | 70.219 | 12 | 3.79E-09 | * |
| DQA1~DQB1 | 34.534 | 8 | 0.001 | * | 78.945 | 12 | 1.38E-10 | * | N/T | N/T | N/T | N/T |
| DPA1~DPB1 | 9.446 | 8 | 6.122 | NS | 4.982 | 7 | 13.906 | NS | N/T | N/T | N/T | N/T |
| All loci ^a^ | NCalc | NCalc | NCalc | NCalc | 23.984 | 3 | 5.29E-04 | * | 12.516 | 2 | 0.025 | * |

Abbreviations: EOMG, early-onset myasthenia gravis and non-thymomatous; *χ*^2^, chi-squared value; d.f., degrees of freedom; *P_adj*, Bonferroni corrected probability (*P*) values; *, statistically significant *P*-values; NS, not significant *P*-values; NCalc, not-calculated; NT, not-tested. ^a^ All loci denotes the *A*~*C*~*B*~*DRB345*~*DRB1~DQA1*~ *DQB1~DPA1*~*DPB1* haplotype in Italians and Norwegians, and the *A*~*C*~*B*~*DRB1~DQB1*~*DPB1* haplotype in the Swedish group.
