# Supplementary material for: Next-Generation Sequencing Identifies Extended HLA Class I and II Haplotypes Associated With Early-Onset and Late-Onset Myasthenia Gravis in Italian, Norwegian, and Swedish Populations
Source: Front Immunol. 2021 Jun 7;12:667336. doi: 10.3389/fimmu.2021.667336 (PMC8215161; doi:10.3389/fimmu.2021.667336)
Supplement: Supplementary file 3 [file Table_3.doc]

**Supplementary Table 3.** Locus-level heterogeneity between LOMG AChR-antibody positive cases and controls

|  | **Italian** | | | | **Norwegian** | | | | **Swedish** | | | |
| --- | --- | --- | --- | --- | --- | --- | --- | --- | --- | --- | --- | --- |
| HLA Locus | *χ*^2^ | d.f. | *P*_adj | Significance | *χ*^2^ | d.f. | *P*_adj | Significance | *χ*^2^ | d.f. | *P*_adj | Significance |
| A | 16.319 | 7 | 0.447 | NS | 7.665 | 9 | 11.933 | NS | 15.739 | 9 | 0.943 | NS |
| C | 4.250 | 7 | 15.011 | NS | 17.312 | 11 | 2.078 | NS | 23.476 | 10 | 0.119 | NS |
| B | 4.654 | 5 | 9.192 | NS | 13.245 | 10 | 4.416 | NS | 24.918 | 10 | 0.072 | NS |
| DRB345 | 20.586 | 8 | 0.167 | NS | 22.998 | 9 | 0.130 | NS | N/T | N/T | N/T | N/T |
| DRB1 | 26.348 | 9 | 0.036 | * | 29.018 | 11 | 0.047 | * | 35.989 | 10 | 0.001 | * |
| DQA1 | 23.240 | 9 | 0.114 | NS | 29.223 | 11 | 0.044 | * | N/T | N/T | N/T | N/T |
| DQB1 | 13.297 | 8 | 2.041 | NS | 31.368 | 11 | 0.020 | * | 26.947 | 9 | 0.019 | * |
| DPA1 | 6.759 | 7 | 9.088 | NS | 5.632 | 7 | 12.249 | NS | N/T | N/T | N/T | N/T |
| DPB1 | 2.726 | 5 | 14.842 | NS | 3.095 | 5 | 14.391 | NS | 11.010 | 6 | 1.145 | NS |
| A~C | 0.825 | 2 | 13.242 | NS | 6.930 | 9 | 13.532 | NS | 19.652 | 7 | 0.083 | NS |
| A~B | 1.772 | 2 | 8.248 | NS | 4.723 | 6 | 12.177 | NS | 13.397 | 7 | 0.819 | NS |
| C~B | 4.222 | 3 | 4.770 | NS | 14.328 | 10 | 3.329 | NS | 23.125 | 8 | 0.042 | * |
| A~C~B | 1.188 | 1 | 5.513 | NS | 7.719 | 7 | 7.518 | NS | 15.795 | 8 | 0.590 | NS |
| B~DRB1 | 0.110 | 1 | 14.794 | NS | 15.682 | 8 | 0.991 | NS | 15.569 | 6 | 0.211 | NS |
| DRB345~DRB1 | 17.106 | 8 | 0.581 | NS | 30.351 | 13 | 0.088 | NS | N/T | N/T | N/T | N/T |
| DRB345~DRB1~DQA1~DQB1 | 5.530 | 7 | 11.911 | NS | 33.082 | 14 | 0.059 | NS | N/T | N/T | N/T | N/T |
| DRB1~DQA1 | 23.175 | 9 | 0.116 | NS | 29.160 | 12 | 0.078 | NS | N/T | N/T | N/T | N/T |
| DRB1~DQB1 | 15.965 | 8 | 0.858 | NS | 34.472 | 13 | 0.021 | * | 37.456 | 12 | 0.002 | * |
| DQA1~DQB1 | 14.200 | 8 | 1.534 | NS | 36.137 | 12 | 0.006 | * | N/T | N/T | N/T | N/T |
| DPA1~DPB1 | 7.841 | 7 | 6.936 | NS | 5.796 | 6 | 9.374 | NS | N/T | N/T | N/T | N/T |
| All loci | NCalc | NCalc | NCalc | NCalc | 11.232 | 3 | 0.221 | NS | 9.184 | 2 | 0.132 | NS |

Abbreviations: LOMG, late-onset myasthenia gravis and non-thymomatous; *χ*^2^, chi-squared value; d.f., degrees of freedom; *P_adj*, Bonferroni corrected probability (*P*) values; *, statistically significant *P*-values; NS, not significant *P*-values; NCalc, not-calculated; NT, not-tested. ^a^ All loci denotes the *A~C~B~DRB345~DRB1~DQA1~* *DQB1~DPA1~DPB1* haplotype in Italians and Norwegians, and the *A~C~B~DRB1~DQB1~DPB1* haplotype in the Swedish group.
